# A rare double Maisonneuve fracture involving concurrent proximal and distal fibula; Weber type A fracture: A case report

**DOI:** 10.1016/j.tcr.2026.101295

**Published:** 2026-01-21

**Authors:** Akiko Sakai, Natsumi Saka, Kentaro Matsui, Kenjin Nishioka, Tomoo Nakagawa, Yoshinobu Watanabe, Hirotaka Kawano

**Affiliations:** a Department of Orthopaedic Surgery, Teikyo University School of Medicine, 2-11-1, Kaga, Itabashi, Tokyo, 173-8605, Japan; b Teikyo Academic Research Center, Teikyo University, 2-11-1, Kaga, Itabashi, Tokyo, 173-8605, Japan

**Keywords:** Maisonneuve fracture, Weber type A fracture, Internal fixation, Nitinol staple

## Abstract

**Background:**

Double Maisonneuve fracture is an extremely rare traumatic condition characterized by simultaneous proximal and distal fibular fractures with syndesmotic disruption. Previously reported cases involved only Weber type B or C distal fibular fractures. To date, no reports have described an associated Weber type A fracture. We report a case of a double Maisonneuve fracture involving a Weber type A distal fibular fracture and describe the clinical course and surgical management.

**Methods:**

A 63-year-old man sustained a left lower leg crush injury involving a Weber type A distal fibular fracture, proximal fibular fracture, medial and posterior malleolar fractures, and tibiofibular diastasis. After initial external fixation, definitive internal fixation was performed on postoperative day 9. The Weber type A fracture was treated using nitinol staples, and fibular length restoration was achieved with a foot distractor. The ankle mortise was stabilized using a one-third tubular plate and trans-syndesmotic screws.

**Results:**

Postoperative imaging confirmed adequate fracture reduction and restoration of the ankle mortise. One year after surgery, radiographs and CT showed complete union and maintained syndesmotic alignment. The patient achieved a Japanese Society for Surgery of the Foot (JSSF) score of 90/100, with full range of motion in both the knee and ankle.

**Conclusions:**

Double Maisonneuve fracture with a Weber type A distal fibular fracture is extremely rare. Accurate diagnosis requires careful palpation of the proximal fibula and imaging of the entire lower leg. Favorable outcomes can be achieved through proper restoration of fibular length and stabilization of the ankle mortise.

## Highlights

• This case represents the first report of a double Masonneuve fracture with Weber type A distal fibula fracture.
• Accurate diagnosis required imaging of both ankle and entire lower leg, as proximal fibular fractures can be easily missed.
• Nitinol staples was effective for the distal fracture, avoiding interference with syndesmotic hardware.
• Restoration of fibular length with a foot distractor and a one-third tubular plate resulted in favorable clinical outcomes.

## Introduction

Maisonneuve fracture (MF) is characterized by fracture of the proximal fibula and an unstable ankle injury, first described in 1840 by the French surgeon Jules Germain Maisonneuve [1]. MF accounts for approximately 5% of all ankle fractures requiring surgery. This uncommon ankle injury is frequently overlooked because proximal fibular fractures may go undetected without a thorough physical examination [2], [3], [4], [5], [6]. Pronation-external rotation is the primary injury mechanism in most cases of MF [7], [8], [9].

Double MF fracture is a variant of MF that involves simultaneous fractures of the proximal and distal fibula, typically accompanied by syndesmotic disruption due to rupture of the distal tibiofibular ligament complex [2]. This injury is recognized as a rare traumatic condition, with limited documentation. To date, three case reports and one case series involving 11 cases have been published [2], [3], [4], [5]. Previously reported distal fibular fractures associated with double MF have been classified as Weber type B or C, with no documented cases of Weber type A distal fibular fractures. Herein, we present the clinical course and result of internal fixation of the double MF with Weber type A distal fibular fracture.

## Case report

**Fig. 1:**
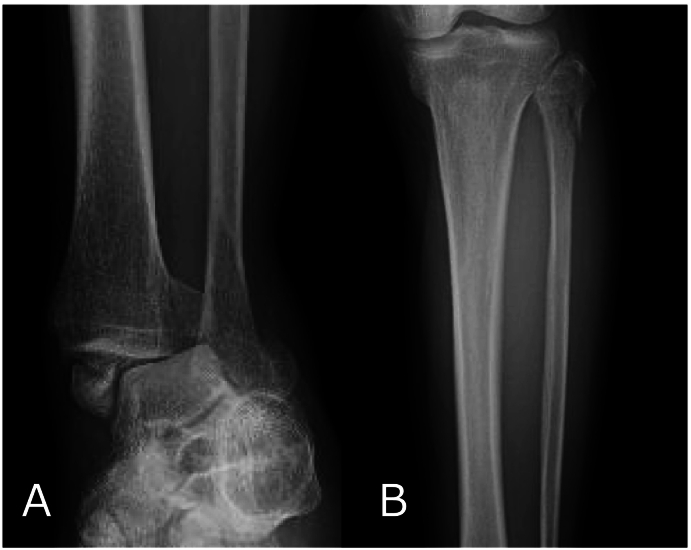
Preoperative X-ray findings. A) Anterior posterior view of the ankle. B) Anterior posterior view of the proximal lower leg.

**Fig. 2:**
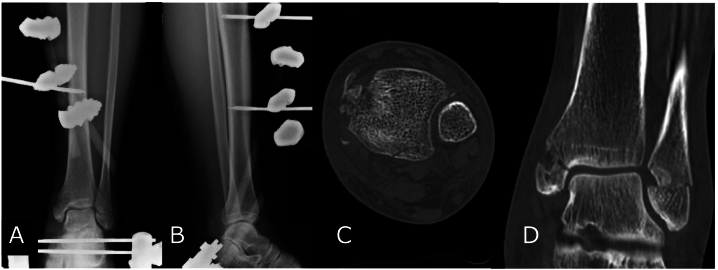
X-rays and CT after the reduction. A) Anterior posterior view of X-ray of the lower leg. B) Lateral view of X-ray of the lower leg. C) Axial view of CT showed posterior malleolar fracture. D) A compression fracture was identified proximal to the Weber type A distal fibular fracture.

The patient was a 63-year-old man brought to our hospital for emergency care after the front wheel of a truck ran over his left lower leg. Physical examination revealed an ankle deformity, abrasions over the medial malleolus, and tenderness around the knee. No circulatory, sensory, or motor impairments were observed. Radiographs and a computed tomography (CT) scan of the ankle and lower leg revealed a Weber type A fracture of the distal fibula, a fracture of the proximal fibula, fractures of the medial and posterior malleoli, and an increased tibiofibular clear space, leading to a diagnosis of ankle joint dislocation fracture. (AO/OTA classification: 44C3.3p + 44A1.3) (Figs. 1A, B, 2A, B, and C). A cartilage defect was also observed near the Weber type A fibular fracture site (Fig. 2D). Due to marked instability of the ankle joint, primary external fixation was performed on the day of the injury, followed by internal fixation on post-injury day 9.

### Surgical technique

**Fig. 3:**
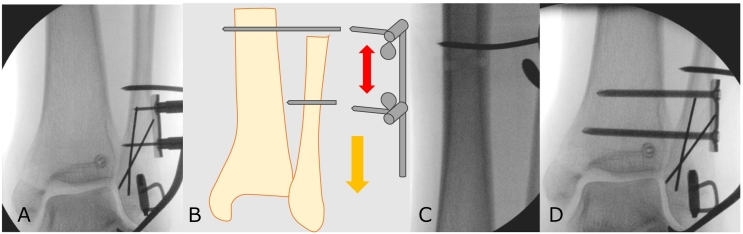
Intraoperative findings and surgical procedures. A) Weber type A fracture was fixed with two nitinol staples. A, B, C) A 2.5-mm Kirschner wire was inserted into the proximal tibia to serve as an anchor, and another wire was placed in the distal fibula. A foot distractor was applied between the two Kirschner wires to restore the fibular length. D) One-third tubular plate and positioning screws were used to fix the syndesmosis.

Surgery was performed under general anesthesia with the patient in the supine position. First, the posterior malleolar fracture was fixed percutaneously using a hydroxyapatite particles/poly l-lactide composite device (OSTEOTRANS®) percutaneously. Next, a skin incision was made along the posterior margin of the fibula, curving slightly anteriorly towards the tarsal sinus, to access the distal fibular fracture. A beta-tricalcium phosphate (OSferion®) was filled in the subchondral bone defect near the Weber type A fracture through the fracture site in order to obtain the articular surface reduction. The Weber type A fracture was then fixed using two nitinol staples (DynaNite®) (Fig. 3A). Third, to obtain the reduction of ankle mortise, fibular length was re-established using a foot distractor. Temporary fixation was achieved with a 2.5 mm Kirschner wire inserted into the proximal tibia as an anchor and another into the distal fibula (Fig. 3A, B, and C). Fibular length was reduced by expanding the distractor using Weber's three indices under fluoroscopic guidance [10]. The length was maintained using a one-third tubular plate, and two screws were inserted from the fibula to the tibia (Fig. 3D). Medical malleolar fracture was fixed using two 3.5 mm cannulated cancellous screws. The proximal fibular fracture was not surgically tested.

### Postoperative course

**Fig. 4:**
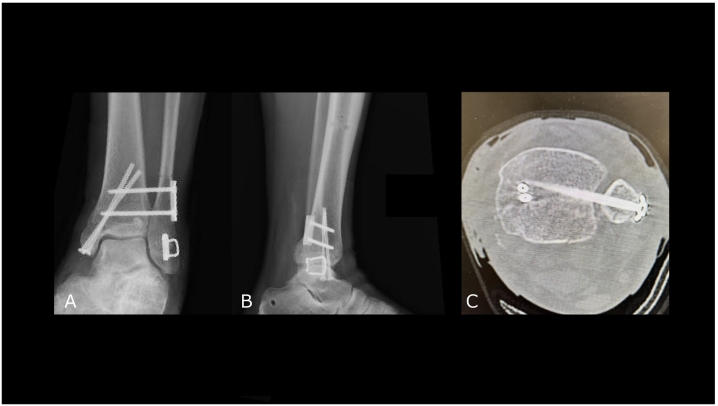
Postoperative findings. A) Anteroposterior X-ray of the ankle. B) Lateral X-ray of the ankle. C) Axial CT view of the distal tibiofibular joint. The proper reduction of the tibiofibular syndesmosis was obtained.

**Fig. 5:**
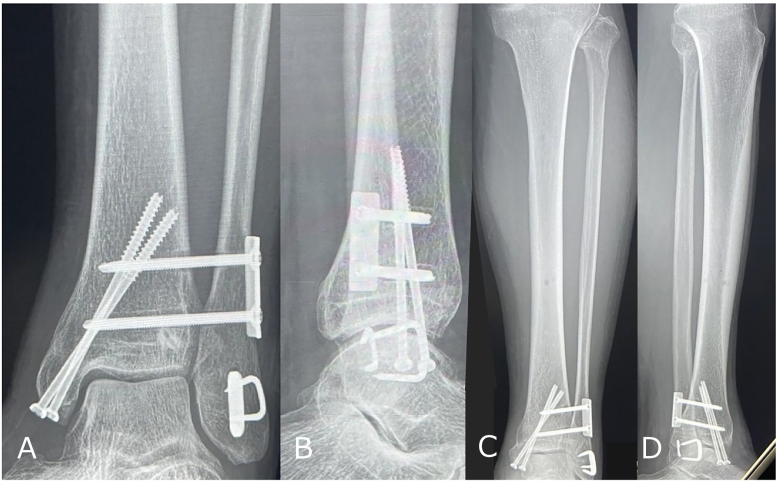
One year postoperative findings. A) Anterior posterior view of the ankle. B) Lateral view of the ankle. C) Anterior posterior view of the lower leg. D) Lateral view of the lower leg.

Postoperative radiographs confirmed satisfactory fracture reduction and restoration of the tibiofibular space (Fig. 4A, B, and C). The patient was instructed to maintain non-weight bearing for two weeks postoperatively with splint immobilization. Partial weight bearing (10 kg) was initiated at three weeks postoperatively, followed by full weight bearing at four weeks. One year postoperatively, radiographs and a CT scan demonstrated union of the proximal and distal fibula with maintained syndesmotic reduction (Fig. 5A, B, C, and D). The patient scored 90/100 on the JSSF (the Japanese Society for Surgery of the Foot) standard rating system [11]. There was no limitation in knee or ankle joint range of motion: knee extension 0/0 degrees, knee flexion 140/140 degrees, dorsiflexion 25/20 degrees, plantarflexion 40/40 degrees.

## Discussion

We encountered a case of double MF with a Weber type A fibular fracture treated using a foot distractor to achieve ankle mortise reduction and fixation with nitinol staples. Although reports of double MF are limited, all previously documented cases of distal fibular fractures associated with MF have been classified as Weber type B or C. To our knowledge, there have been no prior reports of double MF involving a Weber type A fracture [2], [3], [4], [5].

Several mechanisms can be considered as potential etiologies of this injury. In general, MF is caused by pronation-external rotation injury, which is stage III or IV mechanism according to Lauge-Hansen classification [7], [8], [12], [13]. The medial structure is first damaged, which causes fracture of the medial malleolus or rupture of the deltoid ligament, followed by rupture of the anterior inferior tibiofibular ligament (AITFL) or the interosseous ligament (IOL), or interosseous membrane (IOM), which then leads to fracture of the proximal fibula [8]. Widening of the tibiofibular space is associated with external rotation of the fibula [12]. Weber type B and C fibula fractures are caused by pronation-external rotation injury, which is the same mechanism as MF. This could explain why double MF is usually associated with Weber type B or C fibula fracture [14]. In contrast, Weber type A fractures are usually caused by supination and adduction injury [13].We assume that a double MF associated with Weber type A fibular fracture is rare because of the different mechanisms. Although the exact mechanism of this case is unknown, we hypothesize the mechanism of this case as follows: Considering previous reports, as other injury patterns of the double MF, pronation-external rotation should be the main mechanism of the fracture. A significant abduction force caused by compression of the distal fibula by the talus may occur during external rotation, which may result in a Weber type A fracture accompanied by cartilage damage.

Appropriate diagnosis is important for double MF. Three case reports and one case series of 11 cases have been reported [2], [3], [4], [5]. According to these previous reports, more than half of the patients with double MF were misdiagnosed at the initial examination because the proximal fibula fracture was overlooked— a diagnostic challenge that is also common in MF in general [2], [12]. These findings suggest that more cases may have gone unrecognized than previously reported. Tenderness in the proximal lower leg may be missed due to the presence of an ankle fracture identified on radiographs and its severe pain. Therefore, radiographs of the lower leg, both anteroposterior and lateral, should be obtained when an ankle injury was suspected. This report shows that a double MF with a proximal fibular fracture may be suspected not only in Weber type B or C ankle fractures, but also in Weber type A fractures.

Management of double MF follows the general treatment of MF, which is open reduction and internal fixation of the medial and posterior malleoli and stabilization of the syndesmosis [6], [7]. External fixation may be performed if the instability persists. When an anatomical ankle joint was obtained, the postoperative course healed without problems [5], [6], [15].

Malreduction in syndesmotic injuries is associated with poor functional outcome [16]. In a Double MF, where both the proximal and distal fibula are fractured, it is critical to repair and maintain the length of the fibula to achieve an appropriate reduction of the syndesmosis [10]. Various methods of syndesmosis fixation have been employed, including screw fixation, fixation using a tight rope system, and a combination of screws and a plate [17], [18]. Transsyndesmotic screw fixation is commonly used; however, it is associated with the possibility of hardware breakage or discomfort. Tight rope fixation, which does not require screw removal, has recently become more frequently used; however, postoperative loosening has been reported [19]. The addition of a one-third tubular plate with transsyndesmotic screw fixation has been reported to enhance the torsional stiffness of the ankle [17]. In our case, Weber's three indices evaluated through mortise views were used to examine the tibiofibular syndesmosis [10]. The fibular length was carefully reduced using a foot distractor and fixed firmly with a one-third tubular plate and transsyndesmotic screws placed between the tibia and fibula. The patient underwent repair of the fibular length and internal fixation to maintain proper ankle joint alignment, which may have resulted in a good postoperative outcome.

The management of Weber type A fractures varies between studies. Nonoperative treatments were also considered when no instability was found [18]. Several options are used for surgical management, such as screws, tension band (cerclage compression) wiring, or plates [20]. Conventional screws can compress and stabilize fractures when inserted effectively, although there is a risk of screw back-out [20]. The tension band technique is simple in terms of material requirements and is economical, but it has poor resistance to rotation and shear [20]. One-third tubular plates are cost-effective, but anatomical locking plates or hooked plates may be selected for more distal fractures, as they could result in strong internal fixation. However, extensive soft-tissue stripping for the placement of the plate could disrupt bone healing [21]. In clinical practice, the use of nitinol staples is expanding and their usefulness in treating diaphyseal fractures has been demonstrated [22]. This technique effectively captures small bone fragments and provides continuous compression, which may contribute to maintaining stable alignment [23]. Furthermore, its relative simplicity may facilitate a reduction in operative time. In the present case, nitinol staples were used to apply compression for the fixation of Weber type A fracture, offering the advantage of not interfering with the tibiofibular fixation screws.

We report a rare ankle fracture comprising a double MF involving Weber type A fibular fracture, likely resulting from a pronation-external rotation mechanism combined with forceful abduction. Our treatment focused on reducing the fibular length to achieve ankle mortise and maintain position using a one-third tubular plate. Nitinol staples were used to fix the Weber type A fibula fracture, which was advantageous not to interfere with the plate and screws, and to apply a strong compression force. Ultimately, the application of our surgical methods for each fracture was tailored to the specific pathology, which may have contributed to favorable clinical outcomes.

## Conclusion

We encountered a case of double MF with a Weber type A distal fibular fracture. When an ankle fracture is identified, radiographs of both the ankle and the entire lower leg should be obtained to avoid missing a diagnosis of double MF. Careful palpitation of the proximal fibula is also essential. A favorable prognosis may be achieved with appropriate restoration of fibular length and maintenance of the ankle mortise.

## Glossary

MF: Maisonneuve fracture
CT: Computed tomography
AITFL: Anterior inferior tibiofibular ligament
IOL: Interosseous ligament
IOM: Interosseous membrane

## CRediT authorship contribution statement

**Akiko Sakai:** Data curation, Visualization, Conceptualization, Writing – original draft. **Natsumi Saka:** Conceptualization, Writing – original draft. **Kentaro Matsui:** Conceptualization, Writing – review & editing. **Kenjin Nishioka:** Data curation. **Tomoo Nakagawa:** Data curation. **Yoshinobu Watanabe:** Supervision, Writing – review & editing. **Hirotaka Kawano:** Supervision.

## Consent

Written informed consent was obtained from the patients for publication and any images.

## Declaration of Generative AI and AI-assisted technologies in the writing process

During the preparation of this work the authors used ChatGPT in order to improve the phrasing. After using this service, the authors reviewed and edited the content as needed and take full responsibility for the content of the published article.

## Declaration of competing interest

The authors declare the following financial interests/personal relationships which may be considered as potential competing interests: Kentaro Matsui reports a relationship with Arthrex Inc. that includes: speaking and lecture fees and travel reimbursement. Natsumi Saka reports a relationship with Stryker Orthopaedics that includes: funding grants and speaking and lecture fees. Akiko Sakai, Natsumi Saka, Kentaro Matsui, Kenjin Nishioka, Tomoo Nakagawa, Yoshinobu Watanabe, Hirotaka Kawano reports a relationship with Stryker Orthopaedics and Zimmer Biomet that includes: funding grants.

## References

[1] Maisonneuve M. (1840). Recherches sur la fracture du péroné. Arch. Gén. Méd..

[2] Kašper Š., Bartoníček J., Rammelt S., Kamin K., Tuček M. (2022). “Double Maisonneuve fracture”: an unknown fracture pattern. Eur. J. Trauma Emerg. Surg..

[3] Slawski D.P., West C. (1995). Maisonneuve fracture with an associated distal fibular fracture. A case report. Clin. Orthop. Relat. Res..

[4] Hensel K.S., Harpstrite J.K. (2002). Maisonneuve fracture associated with a bimalleolar ankle fracture-dislocation: a case report. J. Orthop. Trauma.

[5] Colenbrander R.J., Struijs P.A.A., Ultee J.M. (2005). Bimalleolar ankle fracture with proximal fibular fracture. Arch. Orthop. Trauma Surg..

[6] Stufkens S.A., van den Bekerom M.P.J., Doornberg J.N., van Dijk C.N., Kloen P. (2011). Evidence-based treatment of maisonneuve fractures. J. Foot Ankle Surg..

[7] Porter D.A., Jaggers R.R., Barnes A.F., Rund A.M. (2014). Optimal management of ankle syndesmosis injuries. Open Access J. Sports Med..

[8] He J.-Q., Ma X.-L., Xin J.-Y., Cao H.-B., Li N., Sun Z.-H., Wang G.-X., Fu X., Zhao B., Hu F.-K. (2020). Pathoanatomy and injury mechanism of typical Maisonneuve fracture: pathoanatomy of Maisonneuve fracture. Orthop. Surg..

[9] Pelton K., Thordarson D.B., Barnwell J. (2010). Open versus closed treatment of the fibula in maissoneuve injuries. Foot Ankle Int..

[10] Futamura K., Baba T., Mogami A., Morohashi I., Kanda A., Obayashi O., Sato K., Ueda Y., Kurata Y., Tsuji H., Kaneko K. (2017). Malreduction of syndesmosis injury associated with malleolar ankle fracture can be avoided using Weber’s three indexes in the mortise view. Injury.

[11] Niki H., Aoki H., Inokuchi S., Ozeki S., Kinoshita M., Kura H., Tanaka Y., Noguchi M., Nomura S., Hatori M., Tatsunami S. (2005). Development and reliability of a standard rating system for outcome measurement of foot and ankle disorders I: development of standard rating system. J. Orthop. Sci..

[12] Bartoníček J., Rammelt S., Kašper Š., Malík J., Tuček M. (2019). Pathoanatomy of Maisonneuve fracture based on radiologic and CT examination. Arch. Orthop. Trauma Surg..

[13] Han S.-M., Wu T.-H., Wen J.-X., Wang Y., Cao L., Wu W.-J., Gao B.-L. (2020). Radiographic analysis of adult ankle fractures using combined Danis-Weber and Lauge-Hansen classification systems. Sci. Rep..

[14] Haraguchi N., Armiger R.S. (2009). A new interpretation of the mechanism of ankle fracture. J. Bone Joint Surg. Am..

[15] Rammelt S., Bartoníček J., Kroker L., Neumann A.P. (2021). Surgical fixation of quadrimalleolar fractures of the ankle. J. Orthop. Trauma.

[16] Sagi H.C., Shah A.R., Sanders R.W. (2012). The functional consequence of syndesmotic joint malreduction at a minimum 2-year follow-up. J. Orthop. Trauma.

[17] Clanton T.O., Matheny L.M., Jarvis H.C., Lewis E.V., Ambrose C.G. (2013). Quantitative analysis of torsional stiffness in supplemental one-third tubular plate fixation in the management of isolated syndesmosis injuries: a biomechanical study: a biomechanical study. Foot Ankle Int..

[18] Wang C., Ma X., Wang X., Huang J., Zhang C., Chen L. (2013). Internal fixation of distal tibiofibular syndesmotic injuries: a systematic review with meta-analysis. Int. Orthop..

[19] Shevate I., Salunkhe R., Pervez F., Pawar P. (2024). A prospective study on fixation of syndesmotic ankle injury: tight rope versus screw fixation. Cureus.

[20] Li Z., Zhou H., Zhao Y., Xia J., Yang Y. (2025). Screw and absorbable suture tension band technique for geriatric Weber type A lateral malleolus fractures. J. Orthop..

[21] Bilgetekin Y.G., Çatma M.F., Öztürk A., Ünlü S., Ersan Ö. (2019). Comparison of different locking plate fixation methods in lateral malleolus fractures. Foot Ankle Surg..

[22] Wu J.C., Mills A., Grant K.D., Wiater P.J. (2019). Fracture fixation using shape-memory (ninitol) staples. Orthop. Clin. North Am..

[23] Sleiman A., Bejcek C., Nestler A., Revelt N., Thuppal S., Mills A., Gardner M. (2023). The history of orthopaedic use of nitinol compression staples. Injury.

